# The consensus from The Chinese Society of Hematology on indications, conditioning regimens and donor selection for allogeneic hematopoietic stem cell transplantation: 2021 update

**DOI:** 10.1186/s13045-021-01159-2

**Published:** 2021-09-15

**Authors:** Xiao-hui Zhang, Jing Chen, Ming-Zhe Han, He Huang, Er-lie Jiang, Ming Jiang, Yong-rong Lai, Dai-hong Liu, Qi-Fa Liu, Ting Liu, Han-yun Ren, Yong-Ping Song, Zi-min Sun, Xiao-wen Tang, Jian-min Wang, De-pei Wu, Lan-ping Xu, Xi Zhang, Dao-bin Zhou, Xiao-jun Huang

**Affiliations:** 1 Peking University People’s Hospital, Peking University Institute of Hematology, Beijing Key Laboratory of Hematopoietic Stem Cell Transplantation, National Clinical Research Center for Hematologic Disease, Beijing, China; 2 Shanghai Children’s Medical Center, Shanghai, China; 3 Institute of Hematology and Blood Disease Hospital, Chinese Academy of Medical Sciences and Peking Union Medical College, National Clinical Research Center for Hematologic Disease, Tianjin, China; 4 First Affiliated Hospital of Zhejiang University, Hangzhou, China; 5 The First Affiliated Hospital of Xinjiang Medical University, Urumqi, China; 6 The First Affiliated Hospital of Guangxi Medical University, Guilin, China; 7 General Hospital of PLA (People’s Liberation Army of China), Beijing, China; 8 Nanfang Hospital of Southern Medical University, Guangzhou, China; 9 West China Hospital, Sichuan University, Chengdu, China; 10 Peking University First Hospital, Beijing, China; 11 Affiliated Cancer Hospital of Zhengzhou University, Henan Cancer Hospital, Zhengzhou, China; 12 The First Affiliated Hospital of University of Science and Technology of China, Hefei, China; 13 The First Affiliated Hospital of Soochow Hospital, National Clinical Research Center for Hematologic Disease, Suzhou, China; 14 Changhai Hospital of Shanghai, Naval Medical University, Shanghai, China; 15 Xinqiao Hospital, Army Military Medical University, Chongqing, China; 16 Peking Union Medical College Hospital, Beijing, China; 17 Peking-Tsinghua Center for Life Sciences, Beijing, China

**Keywords:** Consensus, Allogeneic hematopoietic transplantation, China, Indication, Conditioning regimen, Donor selection

## Abstract

The consensus recommendations in 2018 from The Chinese Society of Hematology (CSH) on indications, conditioning regimens and donor selection for allogeneic hematopoietic stem cell transplantation (allo-HSCT) facilitated the standardization of clinical practices of allo-HSCT in China and progressive integration with the world. There have been new developments since the initial publication. To integrate recent developments and further improve the consensus, a panel of experts from the CSH recently updated the consensus recommendations, which are summarized as follows: (1) there is a new algorithm for selecting appropriate donors for allo-HSCT candidates. Haploidentical donors (HIDs) are the preferred donor choice over matched sibling donors (MSDs) for patients with high-risk leukemia or elderly patients with young offspring donors in experienced centers. This replaces the previous algorithm for donor selection, which favored MSDs over HIDs. (2) Patients with refractory/relapsed lymphoblastic malignancies are now encouraged to undergo salvage treatment with novel immunotherapies prior to HSCT. (3) The consensus has been updated to reflect additional evidence for the application of allo-HSCT in specific groups of patients with hematological malignancies (intermediate-risk acute myeloid leukemia (AML), favorable-risk AML with positive minimal residual disease, and standard-risk acute lymphoblastic leukemia). (4) The consensus has been updated to reflect additional evidence for the application of HSCT in patients with nonmalignant diseases, such as severe aplastic anemia and inherited diseases. (5) The consensus has been updated to reflect additional evidence for the administration of anti-thymocyte globulin, granulocyte colony-stimulating factors and post-transplantation cyclophosphamide in HID-HSCT.

## Background

Allogeneic stem cell transplantation (allo-HSCT) is widely used to treat malignant hematological neoplasms and nonmalignant hematological disorders. The Chinese Blood and Marrow Transplantation Registry Group (CBMTRG) reported that the total annual number of allo-HSCT cases has increased consistently, reaching 9597 cases in 2019, accounting for approximately 14.9% of HSCT cases worldwide [1, 2]. Additionally, 19,798 allo-HSCTs were performed in Europe in 2019, and 9498 allo-HSCTs were performed in the USA in 2019 [3, 4]. Therefore, the standardization of allo-HSCT practices in China would have a major global impact based on the large patient population [2].

The rapid growth of allo-HSCT is a result of the increased availability of alternative donors, especially haploidentical donors (HIDs), ushering in a new era in which “everyone has a donor”. A total of 94% of HID-HSCTs in China follow the “Beijing Protocol”, which includes T-replete HID-HSCT with granulocyte colony-stimulating factor (G-CSF) and antithymocyte globulin (ATG). The administration of post-transplant cyclophosphamide (PT-CY) with or without application of the Beijing protocol has been reported in recent years. Accordingly, HIDs have been the largest source of allo-HSCT donors in China since 2013, and their prevalence among all donors increased to more than 60.1% in 2019 [1]. Other types of donors include matched sibling donors (MSDs) (21.7%), unrelated donors (URDs) (12.8%) and cord blood (CB) donors (5.4%). In contrast, although the frequency of HID-HSCT has increased steadily, reaching approximately 18–20% in Europe and the USA, HIDs account for a small proportion of the donor population compared to URDs, which serve as donors in nearly 50% of allo-HSCT cases [3, 5]. Based on disparities in allo-HSCT practices between China and the Western world, the indications, conditioning regimens, and donor selection methods in China might not be in strict accordance with the current recommendations in the Western world [6, 7]. The first edition of the Chinese consensus on allo-HSCT facilitated the standardization of clinical practices of allo-HSCT in China and progressive integration with the world [8].

There have been new developments since publication of the initial consensus statement. (1) The rapid development of HID-HSCT raised the following questions: “Who is the best alternative donor?” as well as “Who is the best donor?” HID-HSCT has been found to be superior to MSD-HSCT in high-risk leukemia patients and elderly patients with young offspring donors. (2) Patients with lymphoblastic malignancies with a refractory/relapsed (R/R) status are now treated with novel immunotherapies, especially chimeric antigen receptor T-cell (CAR-T) therapies, and then bridged to allo-HSCT. (3) Additional evidence of the superiority of HSCT in patients with intermediate-risk acute myeloid leukemia (AML), positive minimal (or measurable) residual disease (MRD+) favorable-risk AML (CBFb-MYH11+, biallelic mutated CEBPA), and standard-risk Philadelphia chromosome (Ph)-negative acute lymphoblastic leukemia (Ph-ALL) has emerged. (4) There have been an increasing number of studies addressing HSCT in patients with nonmalignant diseases, such as severe aplastic anemia (SAA) and inherited diseases.

Therefore, a panel of experts from the Chinese Society of Hematology (CSH) updated the consensus on indications, conditioning regimens and donor selection processes considering these cutting-edge developments in China and the Western world. These experts represented the most active allo-HSCT centers (approximately 60% of total allo-HSCT cases) in China. The consensus has been updated by iterative, multiple rounds, email-based approach–Delphi consensus protocols. Members of the expert panel first examine the literature after 2018 and provide revised treatment recommendations based on the available evidence. The updated statements were sent to the expert panel, after three rounds of commenting and editing, the panel achieved at least 95% consensus for the current recommendations. The current consensus emphasizes recent advances since 2018, including 87new references that focus on (1) new recommendations based on recent developments and (2) original recommendations supported by new evidence.

## Indications and timing of allo-HSCT

The decision of allo-HSCT is mainly based on the balance between disease progression and transplant-related mortality risk. Recent Chinese studies have suggested that allo-HSCT (including MSD- and alternative donor-HSCT) would benefit specific patient groups by preventing disease progression while maintaining low transplant-related mortality, which supports allo-HSCT over other conventional non-HSCT treatments. Therefore, the current consensus may apply to these patient subgroups, even though it might differ from recommendations from other academic groups, such as the American Society for Transplantation and Cellular Therapy (ASTCT) and the European Cooperative Group for Bone Marrow Transplantation (EBMT) [6, 7].

### Acute myeloid leukemia

AML is the predominant indication for HSCT, accounting for 37% of allo-HSCT cases in China [1]. Allo-HSCT is the standard care option for AML patients classified as having intermediate (int) or adverse (adv) risk (European Leukemia Network, ELN 2017) [9] in first complete remission (CR1). Major progress has been made in recent years in HID-HSCT following the Beijing Protocol. Huang et al. firstly reported that HID-HSCT reduced the relapse rate and improved disease-free survival (DFS) in int/adv-risk AML patients compared to chemotherapy in single center study [10]. In a prospective trial comparing HID-HSCT and chemotherapy as consolidation therapy in 147 patients with int-risk AML CR1 in the absence of MSDs or URDs, HID-HSCT was an independent risk factor for a reduced cumulative incidence of relapse (CIR) (adjusted hazard ratio (HR) 0.161; *p* = 0.001), improved DFS (HR 0.360; *p* = 0.011) and overall survival (OS, HR 0.361, *p* = 0.017) [11]. Yu et al. reported that in a multicenter study of 549 patients with int-risk AML, allo-HSCT had superior CIR, LFS and OS compared with chemotherapy in patients with any positive MRD after 1, 2, or 3 courses of chemotherapy [12].

Addressing the donor source of allo-HSCT for int/adv-risk AML, Wang et al. reported that in a prospective, multicenter study of 450 patients with int/adv-risk AML CR1, and the HID- and MSD-HSCT groups exhibited comparable 3-year CIR (15% vs. 15%, *p* = 0.98), nonrelapse mortality (NRM, 13% vs. 8%, *p* = 0.13), DFS (74% vs. 78%, *p* = 0.34) and OS (79% vs. 82%, *p* = 0.36) [13]. In addition, the results of HID-HSCT following the PT-CY protocol were also comparable to those of MSD-HSCT in AML CR1 patients [14]. Therefore, HID-HSCT and MSD-HSCT are recommended equally as standard care in int/adv-risk AML CR1 patients.

Patients with favorable (fav)-risk AML, including AML due to genetic abnormalities of RUNX1-RUNX1T1, CBFB-MYH11 or biallelic mutated CEBPA (CEBPA^bi+^), may benefit from allo-HSCT during CR1, as it is a risk-directed, MRD-based therapy. The AML05 multicenter trial first demonstrated that MRD-based pretransplant risk stratification may improve the outcome of t (8; 21) AML in CR1; allo-HSCT reduced the CIR (22.1% vs. 78.9%, *p* < 0.0001) and improved DFS (61.7% vs. 19.6%, *p* = 0.001) compared to chemotherapy in the patients for whom major molecular remission (MMR, RUNX1RUNX1 reduction < 3 log units) was not achieved after the second consolidation cycle or who experienced loss of MMR [15]. In addition, similar results were observed in patients with inv (16) AML. Duan et al. reported in single center study that patients with CBFB-MYH11/ABL levels > 0.1% at any time after two consolidation cycles benefited more from allo-HSCT than from chemotherapy in terms of DFS (84.6% vs. 31.4%, *p* < 0.001) [16]. Emerging data also suggested the prognostic values of specific KIT mutations (mainly D816), especially in MRD negative status. Qin YZ suggested allo-HSCT had significantly lower CIR (13.2% vs. 53.2%; *p* < 0.0001) than chemotherapy alone for int-risk t (8; 21) AML patients, defined as KITD816/D820 with MMR or KIT N822/e8/WT patients without MMR, while allo-HSCT could improve OS in high-risk patients of KITD816/D820 without MMR (76.9% vs. 0%, *p* = 0.035) [17]. Similar results suggested the negative impact of KIT D816 mutation in t (8; 21) or inv (16) AML whereas allo-HSCT was superior to auto-HSCT in MRD-negative patients in this subgroup [18, 19]. In CEBPA^bi+^ AML patients with sustained positive MRD after two consolidation cycles, the loss of negative MRD status at any time was the only independent risk factor for CIR, leukemia-free survival (LFS) and OS, while allo-HSCT achieved superior 3-year CIR (0% vs. 52.8%, *p* = 0.006) and LFS (88.9% vs. 47.2%, *p* = 0.027) rates compared to chemotherapy in MRD + patients in single center studies [20, 21]. Among AML patients with nucleophosmin 1 (NPM1) mutation in single center study, those with MRD detected by both multiparameter flow cytometry (FCM) and real-time quantitative polymerase chain reaction (qRT-PCR) were classified as a subgroup with a high relapse risk (46–83%) following chemotherapy [22]; allo-HSCT reduced the CIR and improved DFS in NPM1 + AML in CR1 patients, especially those positive for FMS-like tyrosine kinase 3 internal tandem duplication (FLT3-ITD) [23]. Therefore, MRD-directed risk stratification and the identification of additional risk factors would help guide transplant decisions for patients with fav-AML in CR1 (Table 1).

**Table 1:** Trials comparing allo-HSCT with chemotherapy in acute leukemia

References	Diagnosis	Risk Stratification	CIR	DFS	OS
Lv et al. [11]	Adult Int-AML HID-HSCT vs. CT	Int-AML	11.7% vs. 49.0% *p* < 0.0001	74.3% vs. 47.3%; *p* = 0.0004	80.8% vs. 53.5%; *p* = 0.0001
Zhu et al. [15]	Adult t(8;21)AML-CR1 HSCT vs. CT	High risk: RUNX1-RUNX1 reduction < 3 log units loss of MMR within 6 months	HR 22.1% vs 78.9%	61.7% vs 19.6%	71.6% vs 26.7%
Duan et al. [16]	Adult inv(16)AML-CR1 HSCT vs. CT	CBFB-MYH11/ABL levels > 0.1% at any time after two consolidation cycles	not report	84.6% vs. 31.4%, *p* < 0.001	76.0% vs. 71.0%; *p* = 0.283
Deng et al. [21]	CEBPAbi + AML CR1	sustained positive MRD after two consolidations	0% vs. 52.8%; *p* = 0.006	88.9% vs. 47.2%; *p* = 0.027	88.9% vs. 58.6%;*p* = 0.484
Huang et al. [23]	NPM1 + FLT3 + CT vs. HSCT	Int or fav-AML	not report	HR 0.138 *p* < 0.001	HR 0.173 *p* = 0.001
Chen et al. [106]	NPM-FLT3 + CT vs. HSCT	FLT3-ITD mutant ratio (high and low) FLT3-ITD mutant length (long and short)	FLT3 + HR 0.237 *p* < 0.001; regardless of Ratio/Length	FL3 + HR 0.330 *p* < 0.001; regardless of Ratio/Length	not report
Wang et al. [59]	Ph + ALL HSCT vs. CT + TKIs	white blood cell counts ≥ 30 × 109/L at diagnosis; less than 3 log reduction of BCR-ABL levels after two consolidation cycles	1 risk factor: 23.6 vs. 36.9%, *p* = 0.017; 2 risk factors: 37.5 vs. 100.0%, *p* < 0.001	1 risk factor:62.4 vs. 43.8%, *p* = 0.048; 2 risk factors:56.2 vs. 0%, *p* < 0.001	1 risk factor:76.1% vs. 47.7%, *p* = 0.037; 2 risk factors:51.4% vs. 6.3%, *p* = 0.001
Lv et al. [32]	Adult Ph-ALL HID-HSCT vs. CT	Standard risk-ALL	12.8% vs 46.7%, *p* = 0.0017	80.9% vs 51.1%, *p* = 0.0116	91.2% vs 75.7%, *p* = 0.0408

AML, acute myeloid leukemia; ALL, acute lymphoblastic leukemia; CT, Chemotherapy; HSCT, hematopoietic stem cell transplantation; HID, haploidentical donor; HR, high risk; Int, intermediate risk; DFS, disease-free survival; TKIs, tyrosine kinase inhibitors; Sig, Statistical Significance

The current consensus recommends allo-HSCT as standard care for AML patients with a relatively poor prognosis, such as those in second complete remission or beyond (CR2+), with relapse/refractory (R/R) status, or with therapy-related (t-AML) or myelodysplasia-related changes (AML-MRC), similar to the ASTCT and EBMT guidelines [6, 7]. Yu reported comparable outcomes of 111 cases of refractory AML following MSD- or HID-HSCT in terms of 5-year CIR (32% vs. 23%, *p* = 0.243) and OS (44% vs. 50%, *p* = 0.947) [24].

### Acute lymphoblastic leukemia

ALL accounts for 24% of allo-HSCT cases in China and is the second most prevalent indication [1]. The indications of ALL include two main factors: Ph positivity or negativity and age, stratified as adults and adolescents (age > 14 years) or pediatric patients (age ≤ 14 years) according to the National Comprehensive Cancer Network (NCCN) [25].

Allo-HSCT remains the standard of care for patients with Ph + ALL, even in the era of tyrosine kinase inhibitors (TKIs). Similar results of allo-HSCT have been reported in patients with Ph + ALL following MSD- or HID-HSCT [26, 27]. In addition, the advantages of allo-HSCT are manifested in patients with risk factors. Wang analyzed 91 patients with risk factors, including a white blood cell count ≥ 30 × 10^9^/L at diagnosis; less than a major molecular response, and MMR, defined as a 3-log reduction in BCR-ABL levels after two consolidation cycles. Allo-HSCT was superior to TKIs plus chemotherapy in terms of reducing the CIR (23.6% vs. 36.9%, *p* = 0.017; 37.5% vs. 100.0%, *p* < 0.001) and improving DFS (62.4% vs. 43.8%, *p* = 0.048; 56.2% vs. 0%, *p* < 0.001) in patients with one or two risk factors, respectively [28]. Similarly, in pediatric Ph + ALL patients who failed to achieve MMR, allo-HSCT reduced the CIR and improved DFS compared to imatinib plus pediatric chemotherapy [29].

Allo-HSCT is the standard of care for adult Ph-ALL patients at either high-risk or standard-risk who receive adult chemotherapy regimens. Wang et al. compared allo-HSCT with MSD-HSCT and HID-HSCT in a biological randomized multicenter study of adults with high-risk Ph-ALL in CR1, and there were no differences in the 3-year CIR (18% vs. 24%, *p* = 0.30), transplant-related mortality (TRM) (13% vs. 11%, *p* = 0.84), DFS (61% vs. 60%, *p* = 0.91) or OS (68% vs. 64%, *p* = 0.56) [30]. Allo-HSCT, including HID-HSCT, was also feasible in standard-risk Ph-ALL patients. Han et al. retrospectively investigated the outcomes of allo-HSCT in adults with standard-risk ALL in CR1; patients who received HID-HSCT, MSD-HSCT or matched URD(MUD)-HSCT demonstrated a comparable 5-year CIR (14.8% vs. 21.1% vs. 16.7%, *p* = 0.231), NRM (16.4% vs. 11.6% vs. 19.6%, *p* = 0.162), DFS (68.7% vs. 67.3% vs. 63.7%, *p* = 0.606) and graft-versus-host-disease (GVHD)-relapse-free survival (GRFS; 50.8% vs. 54.9% vs. 52.2%, *p* = 0.847) [31]. In a recent prospective multicenter study of young adults with standard-risk ALL in CR1 in the absence of HLA-matched donors, HID-HSCT was reported to result in a lower 2-year CIR (12.8% vs. 46.7%, *p* = 0.0017) and better 2-year DFS (80.9% vs. 51.1%, *p* = 0.0116) and OS (91.2% vs. 75.7, *p* = 0.0408) than adult chemotherapy [32]. Consequently, HID-HSCT and MSD-HSCT are recommended equally as standard care in patients with high-risk and standard-risk Ph-ALL in CR1. For adolescent and young adult (AYA) patients receiving pediatric-based regimens, the role of allo-HSCT remains to be determined in well-designed clinical trials in the future.

Allo-HSCT in pediatric patients has mainly been performed in patients with high-risk factors, including persistent or recurrent MRD post consolidation or high-risk genetic features [33]. Xue et al. retrospectively analyzed 104 pediatric patients with very high-risk Ph-B-ALL in CR1, and HID-HSCT reduced the CIR (10.9% vs. 46.7%, *p* < 0.001) and improved the LFS rate (81.0% vs. 52.0%, *p* = 0.005) compared to chemotherapy [34]. Xu et al. reported that in 48 consecutive children with high-risk T-ALL, HID-HSCT in CR1 resulted in a lower CIR (19.8% vs. 56.7%, *p* = 0.014) and higher DFS (65.7% vs. 26.0%, *p* = 0.008) than HID-HSCT in non-CR1. In 150 pediatric patients who experienced MRD recurrence (≥ 0.01%), Wang et al. demonstrated that allo-HSCT resulted in a lower 2-year CIR (23.3% vs. 64.0%, *p* < 0.001) and a higher OS rate (88.7% vs. 46.3%, *p* < 0.001) than chemotherapy [35].

Allo-HSCT remains a salvage treatment for relapsed or refractory B-ALL, and an increasing number of patients are being treated with chimeric antigen receptor-modified T (CAR-T) cells or bispecific T-cell engagers (BiTEs) initially and bridged to allo-HSCT. Jiang et al. reported the results of a prospective trial of 58 r/r B-ALL patients who received CD19 CAR T cells. DFS was significantly prolonged by allo-HSCT in the subgroup with either a high level of bone marrow MRD (≥ 5%) or indicators of a poor prognosis [36]. In another prospective trial, reduced intensity conditioning (RIC) with total body irradiation (TBI) allo-HSCT was applied after CR achieved by CD19 or CD22 CAR-T cell treatment. The one-year OS and LFS rates were 87.7% and 73.0%, respectively [37]. Zhang examined 122 R/R ALL patients in a multicenter retrospective study. Pre-transplant MRD-recipients had the lowest CIR and longest LFS compared to the nontransplant group (17.3% vs. 67.2%, *p* < 0.001) and the pretransplant MRD + group (17.3% vs. 65.8%, *p* = 0.006), suggesting that MRD-status is essential for optimizing the outcomes of CAR-T bridging to allo-HSCT [38]. A meta-analysis of 758 R/R ALL patients who received CD19 CAR-T cell therapy indicated that CAR-T cells bridged to allo-HSCT were associated with a lower CIR (HR 0.40, *p* < 0.001) and better DFS (HR 0.20, *p* < 0.001) and OS (HR 0.37, *p* = 0.003) than CAR-T cells alone [39]. In addition to auto-CAR-T cells, donor-derived CAR-T cells are effective in treating r/r B-ALL [40–42].

### Myelodysplastic syndrome

MDS accounts for 8% of allo-HSCT cases in China [1], and allo-HSCT is recommended for advanced MDS (International Prognostic Score System, IPSS Intermediate-2/high-risk) as well as lower-risk MDS with sustained profound cytopenia (neutrophil count < 0.5 × 10^9^/L and/or platelet count < 20 × 10^9^/L).

In a Chinese registry study (CBMTRG) of 454 patients with MDS who underwent allo-HSCT, the 4-year CIR (6%, 7% and 10%, *p* = 0.36) and DFS (58%, 63% and 71%, *p* = 0.14) were comparable between the 3/6 HID-, 4–5/6 HID- and MSD-HSCT patients [43]. Suo et al. also demonstrated that HID-HSCT in pediatric patients with MDS improved 3-year DFS (81.9%) [44].

For advanced MDS patients, as opposed to patients with AML derived from MDS (MDS-AML) or AML-MRC, early referral for HSCT is essential, as no benefit in terms of post-HSCT outcomes was correlated with pre-HSCT cytoreduction [45]. In a study of 228 consecutive advanced MDS patients, Sun et al. reported that cytoreduction did not improve 3-year DFS (70.0% vs. 78.2%, *p* = 0.189) compared to supportive care [46]. In contrast, Wang et al. demonstrated that cytoreduction significantly improved the OS rate (62.2% vs. 20.0%, *p* = 0.013) in MDS-AML patients but not in MDS-EB2 patients (59.2% vs. 62.9%, *p* = 0.991) [47].

### Chronic myelogenous leukemia

Allo-HSCT is no longer the standard of care for chronic myelogenous leukemia (CML) patients in the early chronic phase; consequently, the percentage of allo-HSCTs performed in CML patients in China decreased from 22% in 2008 to less than 2% post 2019 [1]. Additionally, allo-HSCT can be performed in patients with resistance or intolerance to all available first- and second-generation TKIs or with T315I-mutated BCR-ABL. Allo-HSCT remains the standard of care for CML patients in the accelerated phase and blastic crisis. Jiang et al. analyzed 132 CML cases in the accelerated phase in a prospective study, and allo-HSCT showed superior 6-year event-free survival (EFS) (71.8% vs. 39.2%, *p* = 0.008) and OS (83.3% vs. 51.4%, *p* = 0.023). In a retrospective comparison of 83 CML cases in blastic crisis, allo-HSCT significantly improved the 4-year EFS (47.1% vs. 6.7%, *p* < 0.001) and OS (46.7% vs. 9.7%, *p* < 0.001) compared to TKI treatment alone [8].

### Severe aplastic anemia

Although the guidelines of the British Society for Hematology recommended HID-HSCT as only a second-line treatment for refractory SAA after immunosuppressive therapy (IST) failure [48], based on a recent evidence, the current consensus recommends that in addition to HSCT from HLA-matched donors, HID-HSCT can be a first-line option in SAA patients aged less than 50 years and a second-line option in patients aged 51–60 years. The percentage of allo-HSCT for SAA in China increased from 6% in 2008 to more than 13% in 2019, making SAA the third most prevalent indication; more than half of SAA patients received HID-HSCT [1].

In a prospective multicenter clinical trial, Xu et al. examined 101 refractory SAA patients (age ≤ 50 years old) who showed no response to previous IST. HID-HSCT was associated with 3-year failure-free survival (FFS) (86.8% vs. 80.3%, *p* = 0.659) and OS (89.0% vs. 91.0%, *p* = 0.555), similar to MSD-HSCT [49]. Furthermore, in a registry-based comparison study evaluating HID-HSCT as an upfront therapy for SAA, HID-HSCT was associated with similar 3-year FFS (85.0% vs. 89.8%, *p* = 0.413) and OS (86.1% vs. 91.3%, *p* = 0.358) rates compared to MSD-HSCT [50]. Additional evidence from China and the West also supports allo-HSCT from MSDs, URDs and HIDs in both adults and pediatric SAA patients [51–55]. In addition, Liu et al. compared the efficacy of HID-HSCT and IST in 365 patients (age ≤ 55 years old) in a multicenter study and found that more patients in the HID-HSCT group than in the IST group had normal routine blood test results at 6 months post treatment (90.3% vs. 18.8%, *p* < 0.0001) and that these patients had superior FFS (77.8% vs. 48.0%, *p* < 0.0001) and better health-related quality of life (HRQoL) than those treated with IST [56].

### Other nonmalignant hematological diseases

Thalassemia accounts for 5% of allo-HSCT cases in China and is the second most prevalent indication among nonmalignant diseases [1]. He et al. reported the long‐term results of 486 consecutive patients with thalassemia in a multicenter study; 5-year OS (97.4% vs. 92% vs. 94.3% and 97.8%) was comparable among HSCT from MSDs, URDs, HIDs and CB donors, while mismatched URD-HSCT was inferior to MSD-HSCT in terms of 5-year OS (84.6% vs 97.4%, *p* = 0.001) [57].

HID-HSCT is beneficial in paroxysmal nocturnal hemoglobinuria (PNH). Liu et al. demonstrated that HID-HSCT and MSD-HSCT were associated with comparable 3-year OS (86.5% vs. 93.3%, *p* = 0.520) and GRFS (78.3% vs. 92.9%, *p* = 0.250) rates in 40 patients with PNH [58].

Allo-HSCT is the curative treatment for inherited diseases, including inherited metabolic storage diseases (IMDs), such as mucopolysaccharidosis and adrenoleukodystrophy, which accounted for 1% of allo-HSCT cases in 2019 [1]. A registry-based study of mainly CB-HSCT or matched URD-HSCT cases reported that the estimated 3-year OS after allo-HSCT was 84.8%, with 79.4% of patients achieving normal enzyme levels [59]. A pilot study also suggested HID-HSCT as a feasible option for IMDs [60].

In summary, the published evidence suggests that allo-HSCT from MSDs and alternative donors (URDs, HIDs) is associated with equivalent outcomes in patients with indications in China. Therefore, the current consensus does not differentiate recommendations for transplantation based on donor source, which is different from recommendations in Western countries [6, 7].

Systematic, standardized pretransplant risk stratification is important for patients who are eligible for allo-HSCT. Due to disparities in allo-HSCT practices between China and Western countries, the hematopoietic cell transplantation-specific comorbidity index (HCT-CI), EBMT risk score, and disease risk index (DRI) have been validated independently in China [61, 62]. Furthermore, a haploidentical EBMT risk score, which uses the number of HLA disparities instead of donor type, has been developed and validated to predict outcomes in HID-HSCT following the Beijing Protocol [62].

## Recommendation: Indications for and timing of allo-HSCT

### Patients with malignant hematological diseases

1. Acute myeloid leukemia

1.1 AML (non-acute promyelocytic leukemia, non-APL):
  A. AML (non-APL) in CR1
    - Patients with intermediate- or adverse-risk AML according to ELN/NCCN risk stratification [9]
    - Patients who achieve CR1 after > 2 cycles of therapy
    - Patients with AML showing myelodysplasia-related changes or therapy-related myeloid changes
    - Patients with favorable-risk AML showing any of the following features: failure to attain MMR (RUNX1-RUNX1T1 decrease> 3 log) after two consolidation cycles or loss of MMR within 6 months [15, 63]; recurrence of a CBFB-MYH11/ABL level >0.1% at any time after two consolidation cycles [16]; D816 KIT mutation in CBF-AML [17–19]; FCM+ status after two consolidation CEBPAbi+ AML [20]; MRD+ status in NPM1+ AML
  B. AML (non-APL) ≥ CR2
  C. Relapsed or refractory AML (non-APL): allo-HSCT as salvage therapy with individualized conditioning regimens
1.2 APL
  A. Failure to achieve hematological CR by induction therapy
  B. Relapsed APL (molecular, cytogenetic, or hematological relapse) who remain PML-RARA-positive after reinduction

2. Acute lymphoblastic leukemia

2.1 Ph + ALL in adults and adolescents (aged > 14 years old)
  A. Ph + ALL in CR1
  B. Ph + ALL ≥ CR2
  C. Relapsed or refractory Ph + ALL: allo-HSCT as salvage therapy and novel immunotherapies, especially CAR-T therapies, can be applied and then bridged to allo-HSCT [36, 37, 41, 64]
2.2 Ph + ALL in pediatric patients (aged ≤ 14 years old)
  A. Ph + ALL in CR1, especially in patients exhibiting a poor response to prednisone and positive MRD at any time between 4 and 12 weeks after therapy
  B. Ph + ALL ≥ CR2
  C. Relapsed or refractory Ph + ALL: allo-HSCT as salvage therapy, and novel immunotherapies, especially CAR-T therapies, can be applied and then bridged to allo-HSCT
2.3 Ph-ALL in adults and adolescents (aged > 14 years old)
  A. Ph-ALL in CR1: especially in patients with MRD + status or those showing poor-risk factors (aged ≥ 40 years old, high WBC count at diagnosis [100 × 10^9^/L for T lineage and ≥ 30 × 10^9^/L for B lineage], or poor-risk cytogenetics, including Ph + ALL)
  B. Ph-ALL ≥ CR2
  C. Relapsed or refractory Ph-ALL: allo-HSCT as salvage therapy, and novel immunotherapies, especially CAR-T therapies, can be applied and then bridged to allo-HSCT
2.4 Ph-ALL in pediatric patients (aged ≤ 14 years old)
  A. Ph-ALL in CR1:
    - Patients who fail to achieve hematological CR or MRD >1% within 28-30 days
    - Patients who achieve CR with MRD > 0.01% (B-ALL) or MRD>0.1% (T-ALL) post consolidation
    - Patients with MLL/KMT2A+ ALL
  B. Ph-ALL ≥ CR2
  C. Relapsed or refractory Ph-ALL: allo-HSCT as salvage therapy and novel immunotherapies, especially CAR-T therapies, can be applied and then bridged to allo-HSCT

3. Chronic myeloid leukemia:

A. Resistance or intolerance to all available first- and second-generation TKIs
B. T315I-mutated BCR-ABL
C. Accelerated phase status and blastic crisis

4. Myelodysplastic syndromes and myelodysplastic/myeloproliferative neoplasms:

A. IPSS intermediate-2 or high-risk MDS
B. IPSS low-risk or intermediate-1 risk MDS: severe neutropenia or thrombopenia or a high transfusion burden
C. Chronic myelomonocytic leukemia: CMML-specific prognostic scoring system (CPSS) intermediate-2 or high-risk
D. Juvenile myelomonocytic leukemia
E. Atypical chronic myeloid leukemia (BCR-ABL negative), IPSS intermediate-2 or high-risk [65]

5. Myelofibrosis:

- Patients with intermediate-II or high risk according to the Dynamic International Prognostic Scoring System (DIPSS) or DIPSS-plus score [66]

6. Multiple myeloma:

A. Young age and high-risk cytogenetic changes, such as t (4; 14); t (14; 16); 17p-
B. Disease progression after initial auto-HSCT [67]

7. Hodgkin lymphoma:

- Refractory or relapse after auto-HSCT failure [68]

8. Non-Hodgkin lymphoma:

A. Chronic lymphocytic leukemia/small lymphocytic lymphoma (CLL/SLL):
  Allo-HSCT can be considered for young patients under the following conditions in the absence of newly available drugs.
  - Patients who are refractory to available drugs or experience relapse within 12 months
  - Patients who respond to auto-HSCT or available drugs but experience relapse within 24 months
  - Patients with high-risk cytogenetic or molecular factors
  - Patients exhibiting symptoms of Richter syndrome

Others: Allo-HSCT can also be performed in patients with NHL, including follicular lymphoma, diffuse large B-cell lymphoma (DLBCL), mantle cell lymphoma, lymphoblastic cell lymphoma and Burkitt lymphoma, peripheral T-cell lymphoma, and NK/T-cell lymphoma who are refractory, relapsed, or in ≥ CR2. If suitable donors are available, allo-HSCT may also be considered in CR1 for adult patients with mantle cell lymphoma, lymphoblastic cell lymphoma, Burkitt lymphoma, peripheral T-cell lymphoma, NK/T-cell lymphoma, etc.

### Patients with nonmalignant hematological diseases

1. Severe aplastic anemia:
  A. Newly diagnosed SAA:
    Patients (aged ≤ 50 years old) with MSDs can receive MSD-HSCT as a first-line therapy. Pediatric SAA/vSAA patients with ≥ 9/10 loci-matched unrelated donors may also receive allo-HSCT as a first-line therapy. HID-HSCT is recommended for patients without MSDs.
  B. Refractory and/or relapsed SAA:
    Patients (aged ≤ 60 years old) who fail to respond to IST or relapse may undergo
    MSD-, HID-, or MUD-HSCT
2. Paroxysmal nocturnal hemoglobinuria:
  Patients with SAA/PNH who fail to respond to one course of IST or develop clonal evolution of PNH, resulting in MDS/AML [6]
3. Thalassemia:
  Transfusion-dependent severe thalassemia, including severe thalassemia, hemoglobulin E combined with thalassemia, and severe hemoglobulin E disease. Allo-HSCT is recommended before progression to stage 3 in children (aged 2–6 years old).
4. Fanconi anemia:
  Transfusion-dependent Fanconi anemia patients in the moderate cytopenia phase with no poor-risk clonal abnormalities and no MDS/AML [6]
5. Others:
  Congenital immune deficiencies or metabolic diseases, including severe combined immunodeficiency and mucopolysaccharidoses. Allo-HSCT is recommended to be evaluated in clinical trials.

All patients eligible for allo-HSCT should be evaluated using the HCT-CI, Kanofsky or ECOG performance scores, EBMT score or modified EBMT score for HID-HSCT and DRI determination.

## Conditioning regimens

### Standard myeloablative regimens

Standard myeloablative regimen: a modified busulfan (3.2 mg/kg/day, intravenous, i.v., for 3 days) and cyclophosphamide (1.8 g/m2/day, i.v. for 2 days) (mBu/Cy)-based regimen is the most popular regimen in China and is used in up to 59% of allo-HSCT cases. The TBI-based regimen is applied in 12% of allo-HSCT cases, among which two-thirds are TBI + Cy based (Table 2).

**Table 2:** Standard myeloablative regimens

Regimens	Drug	Dose(total)	Schedule(day)	Donor Type
Cy/TBI	Cy	120 mg/kg	− 6, − 5	Allo-HSCT
	f-TBI	12–14 Gy	− 3 to − 1	
Bu/Cy	Bu	16 mg/kg(po)or 12.8 mg/kg(iv)	− 7 to − 4	Allo-HSCT
	Cy	120 mg/kg	− 3, − 2	
Modified BuCy ± ATG	Ara-C	2–4 g/m^2^	− 9	MSD-HSCT
	Bu	9.6 mg/kg(iv)	− 8 to − 6	
	Cy	3.6 g/m^2^	− 5, − 4	
	MeCCNU	250 mg/m^2^(po)	− 3	
	ATG *	4.5 mg	− 4 to − 2	
Modified Cy/TBI ± ATG	Single TBI	770 cGy	− 6	MSD-HSCT
	MeCCNU	250 mg/m^2^(po)	− 3	
	Cy	3.6 g/m^2^	− 5, − 4	
	ATG *	4.5 mg	− 4 to − 2	
Modified BuCy + ATG	Ara-C	4–8 g/m^2^	− 10, − 9	URD,CB,HID-HSCT
	Bu	9.6 mg/kg(iv)	− 8 to − 6	
	Cy	3.6 g/m^2^	− 5, − 4	
	MeCCNU	250 mg/m^2^(po)	− 3	
	ATG	7.5–10 mg	− 5 to − 2	
Modified Cy/TBI + ATG	Single TBI	770 cGy	− 6	URD, HID-HSCT
	MeCCNU	250 mg/m^2^(po)	− 3	
	Cy	3.6 g/m^2^	− 5, − 4	
	ATG	7.5–10 mg	− 5 to − 2	

*Patients age≥40 in MSD-HSCT; Ara-C, cytarabine; allo-HSCT, allogeneic stem cell transplantation; ATG, antithymocyte globulin; Bu, busulfan; Cy, cyclophosphamide; CB, Cord Blood; Flu, fludarabine; IV, intravenous; HID, Haploidentical donor; MSD, matched sibling donor; MeCCNU, Semustine; PO, Oral; TBI, Total body irradiation; URD, unrelated donor

### Reduced intensity regimens

A reduced intensity regimen (RIC) that substitutes (or partially) cyclophosphamide with fludarabine enables more elderly patients (age ≥ 55 years old) and patients with a high risk of comorbidity (such as HCT-CI ≥ 3) to undergo allo-HSCT with acceptable NRM. Bu/Flu-based regimens are applied in 23% of allo-HSCT cases in China. Sun et al. reported that in a prospective single-arm clinical trial of an RIC regimen of HID-HSCT in 50 patients (age ≥ 55) who were conditioned with Bu (3.2 mg/kg/day, intravenous, i.v. for 3 days), Flu (30 mg/m2/day, i.v. for 5 days), Cy (1.0 g/m2/day, i.v. for 2 days) and ATG (2.5 mg/kg/day, i.v. for 4 days), the 1-year NRM, DFS and OS were 23.3, 60.2 and 63.5%, respectively, and were comparable with those of matched patients who received a Bu/Cy/ATG regimen (Table 3) [69].

**Table 3:** Reduced intensity regimens

Conditioning regimen	Drug	Dose (total)	Schedule(d)	Donor type
Flu/Mel	Flu	150 mg/m^2^	− 7 to − 3	Allo-HSCT
	Mel	140 mg/m^2^	− 2, − 1	
Flu/Bu	Flu	150 mg/m^2^	− 9 to − 5	Allo-HSCT
	Bu	8–10 mg/kg (po)	− 6 to − 4	
Flu/Cy	Flu	150 mg/m^2^	− 7 to − 3	Allo-HSCT
	Cy	140 mg/m^2^	− 2, − 1	
Flu/Bu/TT	Flu	150 mg/m^2^	− 7 to − 5	Allo-HSCT
	Bu	8 mg/kg (po)	− 6 to − 4	
	Thiotepa	5 mg/kg	− 3	
RIC-BuFlu ± ATG	Ara-C	2–4 g/m2	− 9	MSD-HSCT
	Bu	9.6 mg/kg (iv)	− 8 to − 6	
	Flu	150 mg/m^2^	− 6 to − 2	
	MeCCNU	250 mg/m^2^	− 3	
	ATG *	4.5 mg	− 4 to − 2	
RIC-mBuCyFlu + ATG	Ara-C	4–8 g/m^2^	− 10, − 9	URD,CB,HID-HSCT
	Bu	9.6 mg/kg (iv)	− 8 to − 6	
	Flu	150 mg/m^2^	− 6 to − 2	
	Cy	2.0 g/m^2^	− 5, − 4	
	MeCCNU	250 mg/m^2^	− 3	
	ATG	7.5–10 mg/kg	− 5 to − 2	

*Patients age≥40 in MSD-HSCT; Flu, fludarabine; Mel, melphan; Cy, cyclophosphamide; Bu, busulfan; Thiotepa; Tespamin; TBI, total body irradiation; Hu hydroxyurea; Ara-C, cytarabine; MeCCNU, Semustine; ATG, antithymocyte globulin; thymoglobuline; allo-HSCT, allogeneic hematological stem cell transplantation; URD, unrelated donor; CB, cord blood; HID, Haploidentical donor

### Intensified conditioning regimens

An intensified conditioning regimen for patients with refractory leukemia may reduce the high malignancy burden and improve outcomes. Yu et al. conducted a prospective study of 278 patients with refractory acute leukemia following sequential intensified conditioning and donor lymphocyte infusion administered in the absence of active GVHD post transplantation to prevent relapse. Both the 5-year OS (46% vs. 42%, *p* = 0.832) and DFS (43% vs. 39%, *p* = 0.665) in the HID and MSD groups were promising [24]. Idarubicin (IDA)-intensified HID-HSCT improves the prognosis of MRD (+ vs. -, CIR 18.9% vs. 11.5%, OS 63.6% vs. 69.6%) [70]. Sequential chemotherapy (FLAG-IDA) followed by fludarabine + busulfan administration was promising, with a 3-year OS rate of 43.8% and an EFS rate of 42.3% [71]. Gao et al. reported that adding decitabine to the Bu/Cy/Flu conditioning regimen resulted in favorable 2-year OS (74% and 86%, respectively) in high-risk and very-high-risk patients with MDS (Table 4) [72].

**Table 4:** Intensified conditioning regimens

Conditioning regimen	Drugs	Dose(total)	Schedule(day)
*International regimens*			
Cy/VP/TBI	Cy	120 mg/kg	− 6, − 5
	Vp16	30–60 mg/m2	− 4
	FTBI	12.0–13.8 Gy	− 3 to − 1
TBI/TT/Cy	FTBI	13. 8 Gy	− 9 to − 6
	TT	10 mg/kg (po)	− 5, − 4
	Cy	120 mg/kg	− 6, − 5
Bu/Cy/MEL	Bu	16 mg/kg (po)	− 7 to − 4
	Cy	120 mg/kg	− 3, − 2
	Mel	140 mg/m2	− 1
*Chinese regimens*			
TBI/VP16	Flu	150 mg/m2	− 10 to − 6
	Ara-C	5–10 g/m2	− 10 to − 6
	TBI	9 Gy	− 5, − 4
	Cy	120 mg/kg	− 3, − 2
	Vp16	30 mg/kg	− 3, − 2
Bu/Cy/IDA	IDA	45 mg/m2	− 11 to − 9
	Bu	9.6 mg/kg(iv)	− 6 to − 4
	Cy	3.6 g/m2	− 3, − 2
Bu/Flu/IDA	Flu	150 mg/m2	− 21 to − 17
	Ara-C	5 g/m2	− 21 to − 17
	IDA	30–36 mg/m2	− 17 to − 15
	Flu	150 mg/m2/day	− 7 to − 3
	Bu	9.6 mg/kg(iv)	− 5 to − 3
Dec/Bu/Cy/Flu	Decitabine	100 mg/m2	− 9 to − 5
	Ara-C	6 g/m2	− 9 to − 7
	Bu	9.6 mg/kg(iv)	− 9 to − 7
	Flu	90 mg/m2	− 6 to − 4
	Cy	80 mg/kg	− 3, − 2

Bu, busulfan; Cy, cyclophosphamide; CsA,cyclosporine; IDA, Idarubicin; Flu, fludarabine, IV, intravenous; VP16, etoposide TBI, Total body irradiation

### Graft-versus-host disease prophylaxis in conditioning

Intensive GVHD prophylaxis, including cyclosporine (CsA), methotrexate (MTX), mycophenolate mofetil (MMF) and ATG, is part of the conditioning regimen. Two randomized controlled trials helped to validate the ideal ATG dosage for GVHD prophylaxis in HID-HSCT patients. In the first trial, 224 patients were randomly assigned (1:1) to receive 10 mg/kg (ATG-10) or 6 mg/kg (ATG-6) ATG; ATG-6 administration resulted in higher incidence rates of grade III-IV acute GVHD (16.1% vs. 4.5% *p* = 0.005) and 5-year moderate-to-severe chronic (c)GVHD (56.3% vs. 30.4%, *p* < 0.0001) than ATG-10 administration [73]. Since ATG-6 was associated with a high risk of GVHD, Wang et al. recently conducted a multicenter randomized trial including 408 patients to compare outcomes associated with 7.5 mg/kg and 10 mg/kg ATG administration. They found a lower rate of infection-related mortality and similar rates of grade II-IV acute (a)GVHD (27.1% vs. 25.4%, *p* = 0.548), 2-year cGVHD (34.6% vs. 36.2%, *p* = 0.814), 3-year OS (69.5% vs. 63.5%, *p* = 0.308) and DFS (62.2% vs. 60.3%, *p* = 0.660), suggesting that ATG-7.5 administration might be preferred in HID-HSCT following the Beijing Protocol [74, 75]. Chang et al. investigated the outcomes of 263 patients (aged > 40 years old) who remained at high risk of aGVHD following MSD-HSCT in a randomized controlled trial; the patients were randomly assigned to the ATG group (4.5 mg/kg thymoglobulin plus CsA + MTX + MMF) or the control group (CsA + MTX + MMF). ATG administration reduced the rates of grade II-IV aGVHD (13.7% vs. 27.0%, *p* = 0.007) and overall chronic GVHD (27.9% vs. 52.5%, *p* < 0.001) and improved 3-year GRFS (38.7% vs. 24.5%, *p* = 0.003) compared with the control [76]. ATG administration was also associated with a higher IST-free survival rate, a lower NRM rate, and superior OS and GRFS rates in URD-HSCT patients [77].

Recently, PTCy-based regimens have also been applied in China, although they are applied in less than 5% of HID-HSCT cases. Tang et al. compared the outcomes of HID-HSCT following either the Beijing protocol or PTCy protocol with regard to hematologic malignancies in 220 patients with the nested case-pair method in a Chinese registry study (CBMTRG); the Beijing protocol was associated with a higher incidence of 30-day neutrophil engraftment (96.6% vs. 88.6%, *p* = 0.001), higher incidence of 90-day platelet engraftment (94.2% vs. 84.1%, *p* = 0.04), lower 3-year NRM (12.0% vs. 27.3%, *p* = 0.008), longer DFS (74.3% vs. 61%, *p* = 0.045) and OS (78.3% vs. 65.2%, *p* = 0.039) than the PTCy protocol, while the results in those with aGVHD and cGVHD were comparable [78]. Xu investigated 100 patients with SAA who underwent HSCT following the Beijing protocol or PTCy protocol in a Chinese registry study; the incidence rates of grade II-IV aGVHD (15.0% vs. 32.5%, *p* = 0.111), cGVHD (27.9% vs. 20.7%, *p* = 0.699), FFS (83.8% vs. 87.3%, *p* = 0.679) and OS (89.1% vs. 88.5%, *p* = 0.972) were comparable [79]. Furthermore, adding low-dose PTCy to the standard Beijing protocol appears to be a potential strategy for patients with mother or collateral donors who have a high risk of aGVHD [80, 81]. Wang et al. conducted a prospective study including 239 patients who were treated with ATG with or without low-dose PTCy (14.5 mg/kg on days + 3 and + 4). The rates of grade III-IV aGVHD and NRM in the ATG-PTCy cohort were significantly reduced compared with those in the ATG group (5% vs. 18%, *p* = 0.003; 6% vs. 15%, *p* = 0.045). The ATG-PTCy group exhibited improved GRFS (63% vs. 48%, *p* = 0.039), while the 2-year CIR (13% vs. 14%, *p* = 0.62) and OS rate (83% vs. 77%, *p* = 0.18) were comparable [82]. In another prospective study, patients received low-dose ATG (5 mg/kg) and low-dose PTCy (50 mg/kg) for GVHD prophylaxis following HID-HSCT. The rates of grades II-IV and III-IV aGVHD were 19.4% and 6.9%, respectively. The 1-year CIR, LFS, and OS rates were 25.1%, 59% and 78.4%, respectively [83]. Therefore, adding low-dose PTCy to the Beijing protocol might be a potentially effective strategy for patients with a high risk of aGVHD in HID-HSCT.

## Recommendation: Conditioning regimens

The myeloablative and reduced intensity regimens are defined according to the ASTCT [84].

### Patients with malignant hematological diseases

1. Patients with leukemia/MDS:

1.1 Standard myeloablative regimen: MAC regimens include traditional total body irradiation plus cyclophosphamide (TBI/Cy), busulfan plus cyclophosphamide (Bu/Cy), and associated modified regimens (Table 2).
1.2 Reduced intensity regimen: Fludarabine-containing regimens are commonly used (Table 3).
1.3 Intensified regimen: Intensified regimens generally include the addition of a drug, such as idarubicin, etoposide, fludarabine, melphalan, decitabine or TBI, to a standard conditioning regimen. It is primarily used in refractory patients or those with relapsed malignancy. As most intensified regimens have not been evaluated in multi-center or registry-based studies, it should be chosen with caution according to the center experiences (Table 4).

ATG (rabbit anti-thymocyte globulin, Sangstat, Lyon, France) is used in a dose range of 7.5–10 mg/kg for HID-HSCT, 4.5–10 mg/kg for URD-HSCT, and 4.5 mg/kg for MSD-HSCT [73–77]. Adding low-dose PTCy (14.5 mg/kg + 3, + 4 or 50 mg/kg + 3) to the Beijing protocol might be appropriate in patients at high risk of GVHD [82, 83].

The optimal conditioning regimen for a patient should be selected based on the type and status of the disease, comorbidities, underlying conditions, and donor type. For example, a standard-intensity conditioning regimen is used in younger patients (younger than 55 years old), and RIC regimens are used in patients older than 55 years or patients with poor organ function (HCT-CI ≥ 3) [69].

2. Patients with malignant hematological diseases other than leukemia/MDS:
  Conditioning protocols, such as carmustine + etoposide + cytarabine + melphalan (BEAM), fludarabine/melphalan (Flu/Mel) or fludarabine/busulfan (Flu/Bu), can generally be used in patients with MM or NHL (Table 5). MAC regimens, such as the BuCy, TBICy, or modified BuCy regimens, may also be used in patients with MM or NHL.

**Table 5:** Conditioning regimens for multiple myeloma and lymphoma

Conditioning regimen	Drug	Dose	Schedule(day)	Indications
BEAM	BCNU	300 mg/m^2^	− 6	Lymphoma
	Vp16	800 mg/m^2^	− 5 to − 2	
	Ara-C	800 mg/kg	− 5 to − 2	
	Mel	140 mg/m^2^	− 1	
Flu/MEL	Flu	150 mg/m2	− 7 to − 3	Multiple myeloma
	Mel	140 mg/m^2^	− 2, − 1	
	Bortizomib			
Flu/Bu	Flu	150 mg/m^2^	− 10 to − 6	Multiple myeloma
	Bu	6.4 to 9.6 mg/kg(po)	− 7 to − 4	

BCNU, carmustine; VP16, etoposide; Ara-C, cytarabine; Mel, melphan; Flu, fludarabine; Bu, busulfan; allo-HSCT, allogeneic hematological stem cell transplantation

### Patients with nonmalignant hematological diseases

1. SAA: The Cy-ATG regimen can be used for HLA-matched sibling transplantation, and the FluCy-ATG regimen can be used for unrelated transplantation. The most commonly used regimen in China is the BuCyATG protocol, followed by T-replicate HID-HSCT (Table 6).
2. Thalassemia major: Intensified conditioning regimens instead of standard conditioning regimens should generally be used in patients with thalassemia [57, 85].
3. Fanconi anemia: The FluCy-ATG regimen (Flu 150 mg/m2, Cy 5–20 mg/kg/day × 4 days, and rabbit ATG 10 mg/kg) with or without low-dose TBI may be used for alternative donor transplantation.

**Table 6:** Conditioning regimens for severe aplastic anemia

Conditioning Regimen	Drug	Dose(total)	Schedule(day)	Donor type
Cy-ATG	Cy	200 mg/kg	− 5 to − 2	MSD/URD
	ATG	10 mg/kg	− 5 to − 2	
FluCy-ATG	Flu	120 mg/m^2^	− 5 to − 2	URD/HID
	Cy	90–120 mg/kg	− 3, − 2	
	ATG	10 mg/kg	− 5 to − 2	
Modified BuCyATG [49, 50]	Bu	6.4 mg/kg (iv.)	− 7, − 6	HID
	Cy	200 mg/kg	− 5 to − 2	
	ATG	10 mg/kg	− 5 to − 2	
BuFluPTCy [79]	Bu	6.4 mg/kg (iv.)	− 8, − 7	HID
	Flu	200 mg/m^2^	− 8 to − 4	
	Cy	29 mg/kg	− 3, − 2	
	Cy	120 mg/kg	+ 3, + 4	

ATG, antithymocyte globulin; Bu, busulfan; Cy, cyclophosphamide; Flu, fludarabine; HID, Haploidentical donor; MSD, matched sibling donor; URD, unrelated donor; PT-CY, post-transplantation cyclophosphamide

## Donor selection and graft source

**Fig. 1:**
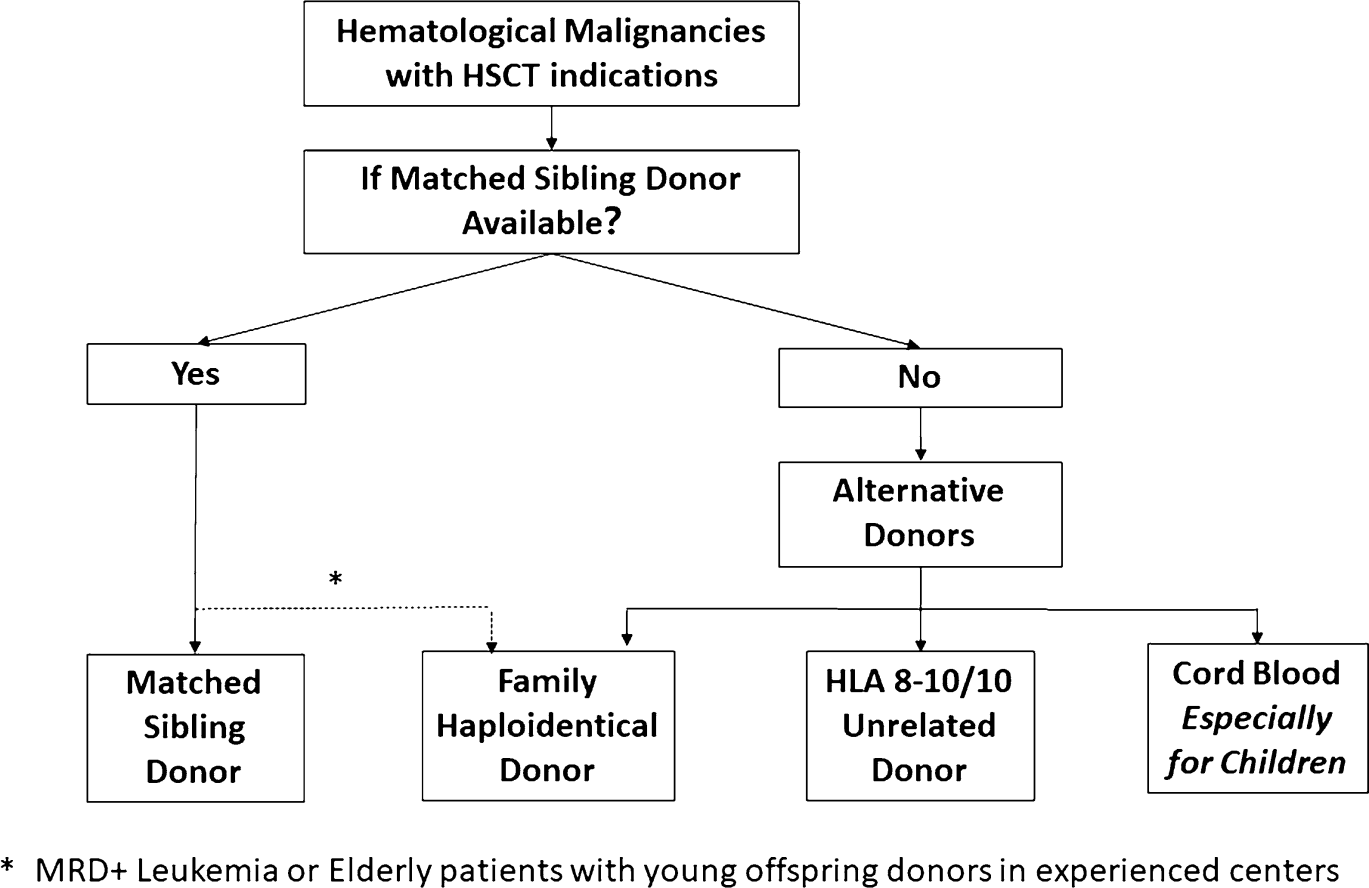
Algorithm for hematological malignancies

**Fig. 2:**
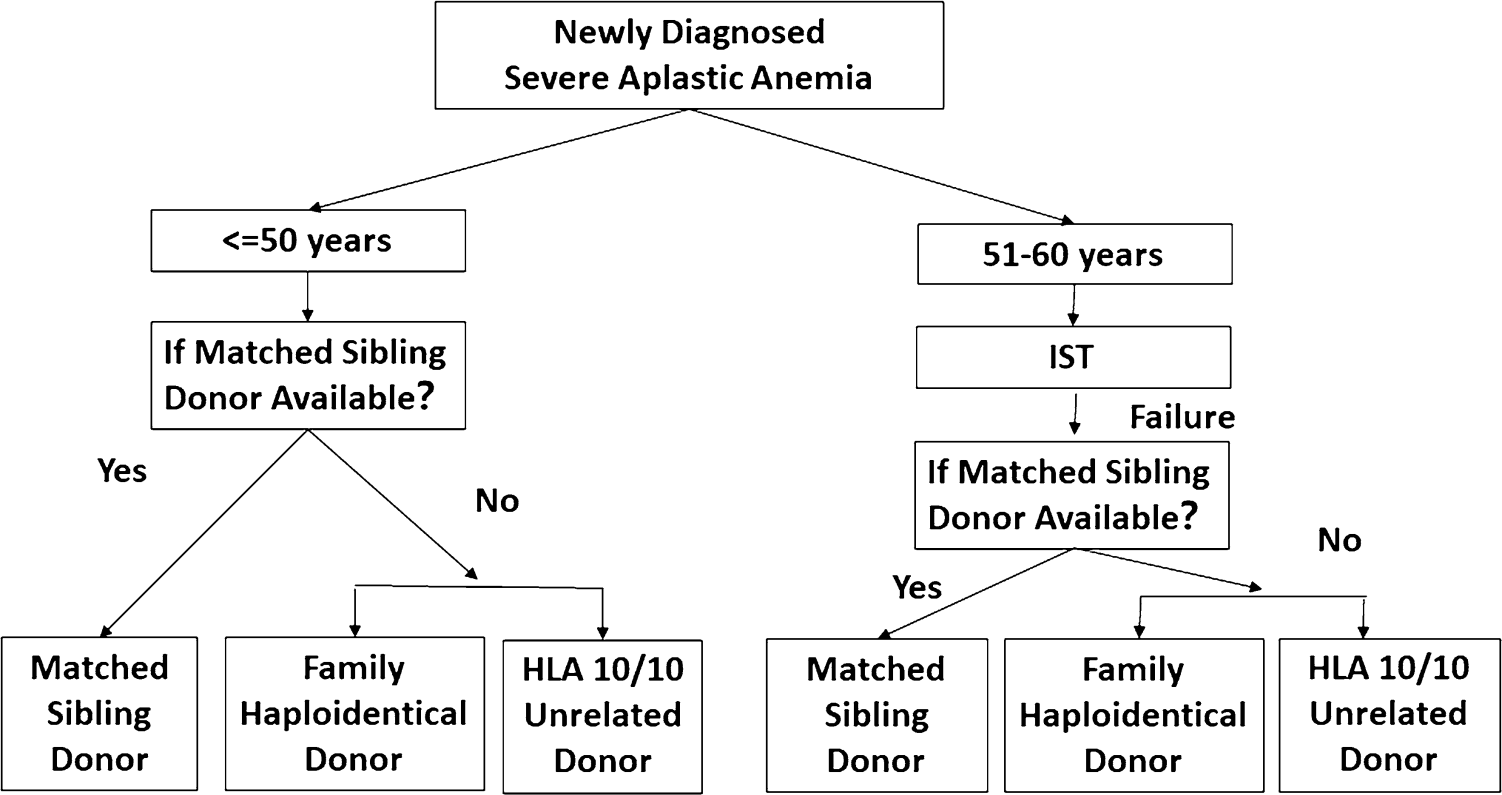
Algorithm for severe aplastic anemia

MSDs are generally preferred for allo-HSCT, with HIDs, URDs, and CB donors as alternatives. HID-HSCT might be superior to MSD-HSCT in high-risk leukemia patients and elderly patients with young offspring donors. Therefore, the ideal donor should be identified based on factors such as recipient condition (high relapse features, refractory or relapsed status, age, and performance status), characteristics of the donor, and experience of the transplantation center (Figs. 1, 2).

### Haploidentical donors

HID-HSCT is associated with a clinical outcome similar to that of MSD- or MUD-HSCT for the treatment of AML, ALL, MDS, and SAA.

The advantages of HIDs include the following: (1) almost all patients can be matched with an HID in an appropriate timeframe; (2) an HID is more suitable for urgent allo-HSCT, especially during the coronavirus disease 2019 (COVID-19) pandemic [86]; (3) re-donation is feasible for further cellular therapy, especially in high-risk relapsed patients; (4) bone marrow and/or peripheral stem cells may be obtained based on clinical condition[87]; and (5) HID-HSCT is associated with a lower incidence of relapse than MSD-HSCT in high-risk hematological malignancy patients [88–92]. It should be noted that the incidence of GVHD is still higher in HID-HSCT than in MSD-HSCT patients.

Donor-specific anti-HLA antibodies (DSAs) play a role in the search for HIDs. Chang et al. focused on the relationship between DSAs and primary graft failure (GF) after HID-HSCT and designed a prospective study with randomly assigned training and validation sets; the results indicated that a median DSA fluorescence intensity (MFI) ≥ 10,000 was significantly correlated with primary graft failure (GF) after HID-HSCT (*p* < 0.001), and HID-HSCT should be avoided in such patients [93]. Rituximab for desensitization might overcome the negative effect on primary poor graft function (PGF) in patients with an MFI between 2000 and 10,000 [94].

**Fig. 3:**
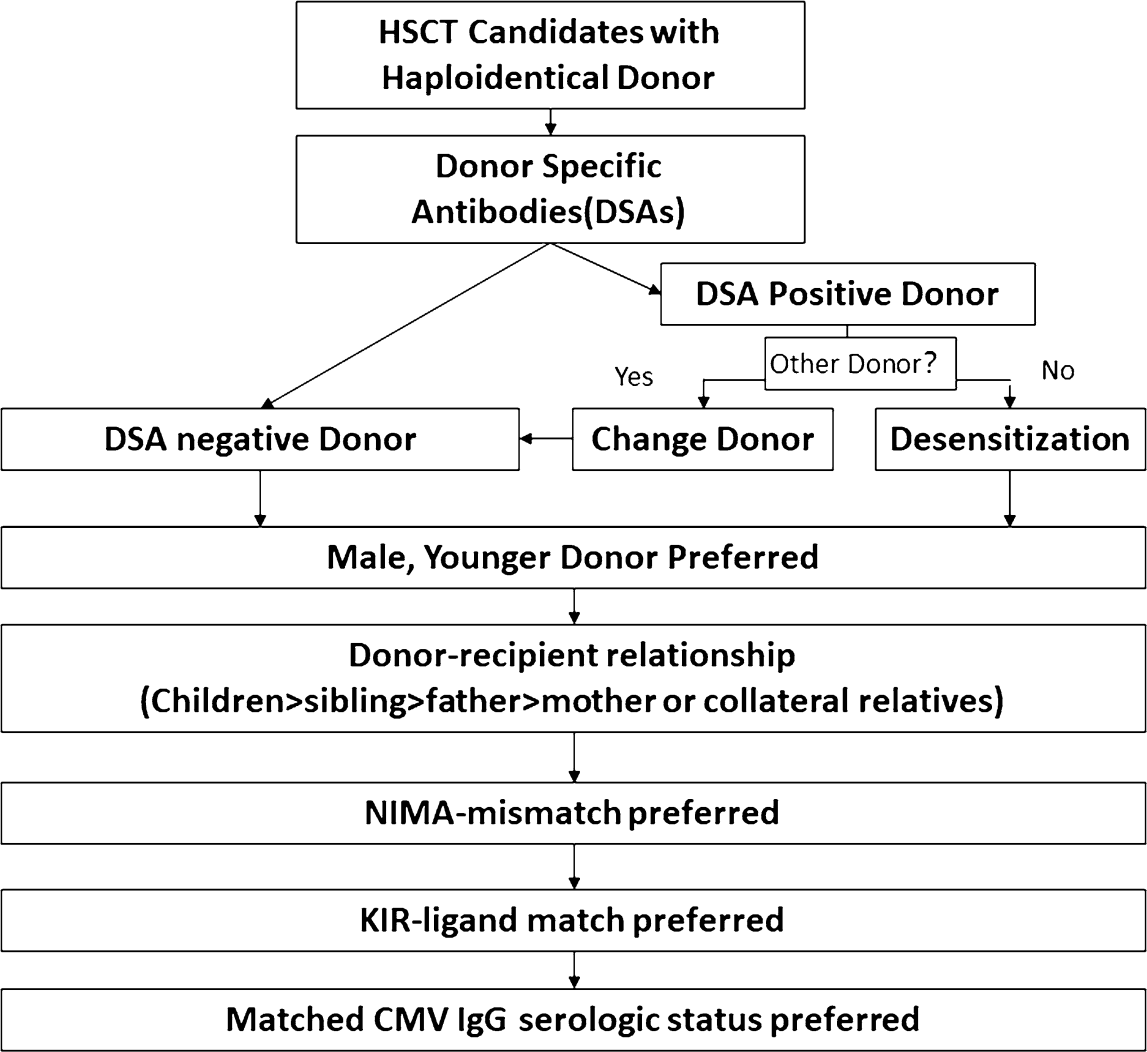
Algorithm for HIDs in hematological malignancies

Based on 1210 consecutive transplant cases uniformly treated with the Beijing protocol, Wang et al. proposed a rank order for choosing an HID for hematological disease patients: 1) young donors, 2) male donors, and 3) noninherited maternal antigen-mismatched donors [81]. Additionally, in a recent multicenter study based on a registry database including 381 patients with SAA, aGVHD, or cGVHD, OS and FFS were comparable among recipients with grafts from fathers, mothers, siblings, or children; all were suitable HIDs for patients with SAA [95]. In addition, there are discrepancies in donor-recipient cytomegalovirus (CMV) serostatus matching and killer immunoglobulin-like receptor (KIR) ligand matching algorithms for HID selection between China and Western countries [96] (Fig. 3).

### Unrelated donors

Clinical outcomes of URD-HSCT have been shown to be similar to those of MSD-HSCT and HID-HSCT in both hematological malignancy and nonmalignant hematological disease patients. Luo et al. compared 305 patients with hematological malignancies who received T-cell-replete HSCT from MSDs, URDs and HIDs, and the 5-year DFS rates were comparable (63.6% vs. 58.4% vs. 58.3%, *p* = 0.574) [97]. Zhang et al. investigated 85 patients with SAA, and similar 3-year OS rates were observed in those who underwent MSD-, URD- and HID-HSCT (92.1% vs. 100% vs. 86.7%, *p* = 0.481) [98]. There have been more than 10,000 donations from the China Marrow Program, and URD-HSCT accounts for 13% of allo-HSCTs. The application of URD-HSCT might be limited by several factors, such as the probability of finding an appropriate donor, urgent transplantation needs, redonation for novel cellular therapies, and the COVID-19 pandemic.

### Cord blood donors

The outcome of CB-HSCT is comparable to that of URD-HSCT or HID-HSCT in mainly children with malignant hematological diseases; CB-HSCT accounts for approximately 5% of allo-HSCTs. Mo et al. compared CB-HSCT and HID-HSCT in 129 children with high-risk ALL, and the outcomes were comparable in terms of the 2-year CIR (24.1% vs. 16.1%, *p* = 0.169), NRM (18.8% vs. 12.8% *p* = 0.277) and OS (69.6% vs. 82.0%, *p* = 0.071) [99]. Tong et al. compared CB-HSCT without ATG administration with URD-HSCT in adult patients in a multicenter retrospective study. CB-HSCT was associated with a lower 3-year cGVHD rate (20.4% vs. 50.0% *p* < 0 0.001), higher 3-year GRFS (54.4% vs. 39.4% *p* = 0.04), and comparable 3-year OS (61.2% vs. 60.9%, *p* = 0.96) and DFS (56.5% vs. 55.5%, *p* = 0.86) compared to URD-HSCT [100]. Currently, single-dose CBT is mainly used in pediatric patients.

### Who is the best allo-HSCT donor?

Although MSDs are generally the preferred choice for allo-HSCT, MSDs might not always be the best allo-HSCT donor for a patient with hematologic malignancy. Wang et al. reported that in a prospective data set of 1199 consecutive subjects, a higher donor/recipient age ratio, female-to-male transplantation, and donor-recipient ABO major-mismatch transplantation were major risk factors for NRM and should be considered priorities over HLA disparity [101]. The benefit of HID-HSCT over MSD-HSCT in elderly patients with young offspring donors was investigated in another multicenter study. Acute leukemia patients (aged ≥ 50 years) undergoing HID- and MSD-HSCT were 1:1 matched for analysis. HID-HSCT was associated with lower three-year NRM (9% vs. 26%, *p* = 0.023), a lower CIR (6% vs. 17%, *p* = 0.066) and higher OS (85% vs. 58%, *p* = 0.003) and DFS (85% vs. 56%, *p* = 0.001) rates than MSD-HSCT. These results might indicate that a young offspring donor is preferred over an older MSD for patients > 50 years old [102].

HID-HSCT might exert a stronger graft-vs-leukemia (GVL) effect and result in better outcomes than MSD-HSCT. Ex vivo experiments showed that cytotoxic T lymphocytes from the HID-HSCT group showed a superior GVL effect [103]. Accordingly, in several AML subgroups, HID-HSCT was found to be superior to MSD-HSCT in reducing the relapse rate and/or improving LFS. Chang et al. reported that pre-HSCT MRD + AML patients receiving HID-HSCT had a lower CIR (19% vs. 55%, *p* < 0.001) and longer LFS (74% vs. 33%, *p* < 0.001) than those receiving MSD-HSCT [88]. Yu et al. demonstrated that patients with ELN 2017 adverse-risk AML in CR1 who underwent HID-HSCT had a lower cumulative incidence of post-HSCT-positive MRD (18% vs. 42%, *p* < 0.001) and longer 3-year GRFS (63% vs. 43%, *p* = 0.035) than their counterparts [91]. Zheng et al. investigated 179 children with high-risk AML, and the CIR in the HID-HSCT group was significantly lower than that in the MSD-HSCT group (39.1% vs. 16.4%, *p* = 0.027) [92]. In addition, in ALL patients with a high risk of relapse, HID-HSCT was associated with a lower 3-year CIR (23% vs. 47%, *p* = 0.006) and longer LFS (65% vs. 43%, *p* = 0.023) and OS (68% vs. 46%, *p* = 0.039) than MSD-HSCT in a phase III randomized trial [89]. In another retrospective study of Ph + ALL with positive pre-MRD, HID-HSCT was associated with a lower 4-year CIR (14.8% vs. 56.4%, *p* = 0.021) and higher 4-year LFS rate (77.7% vs. 35.9%, *p* = 0.036) than MSD-HSCT [90] (Table 7).

**Table 7:** Trials comparing HSCT with haploidentical donor and matched sibling donor

References	Diagnosis	aGVHD II-IV	cGvHD	CIR	LFS or GRFS	OS
Chang et al. [88]	Adult FCM MRD + AML HID vs. MSD	HID: 28–36% MSD: 5–7%	HID: 70–73% MSD: 41–66%	19 vs. 55%, *p* < 0.001	74 vs. 33%, *p* < 0.001	83 vs. 38%, *p* = 0.001
Yu et al. [91]	Adult High-risk AML CR1 HID vs. MSD	HID: 40% MSD: 46%	HID: 39% MSD: 51%	14 vs. 24%, *p* = 0.101	LFS 71 vs. 66%, *p* = 0.579 GRFS 64 vs. 43%; *p* = 0.035	72 vs. 68%, *p* = 0.687
Zheng et al. [92]	Children High-risk AML CR1 HID vs. MSD	HID: 35% MSD: 13%	HID: 35% MSD: 14%	50.0 vs. 9.2%, *p* = 0.001	81.2 vs. 50.0%, *p* = 0.021	81.5 vs. 68.8%; *p* = 0.196
Guo et al. [103]	MRD + AML-ETO HID vs. MSD	Not report	Not report	14 vs. 25%; *p* = 0.036	68 vs. 48%;*p* = 0.026	70 vs. 50%; *p* = 0.062
Chang et al. [89]	Adult FCM MRD + ALL HID vs. MSD	HID: 21% MSD:23%	HID: 41% MSD: 48%	23 vs. 47%, *p* = 0.006	65 vs. 43%, *p* = 0.023	68 vs. 46%, *p* = 0.039
Li et al. [90]	Adult MRD + Ph + ALL HID vs. MSD	HID: 22.0% MSD:23.8%	HID: 38.5% MSD: 38.3%	14.8 vs. 56.4%, *p* = 0.021	77.7 vs. 35.9%, *p* = 0.036	80.5 vs. 35.9%, *p* = 0.027
Gao et al. [26]	Adult and children Ph + ALL HID vs. MSD	HID:51.1% MSD:25.7%	HID:48.9% MSD:25.7%	19.1 vs 44.8%, *p* = 0.036	59.5 vs 45.7%, *p* = 0.118	63.8 vs 62.6%, *p* = 0.743
Wang et al. [102]	Elderly AL HID with young donor vs. MSD	HID: 35% MSD:26%	HID: 24% MSD: 37%	6 vs. 17%; *p* = 0.066	85 vs. 56%; *p* = 0.001	85 vs. 58%; *p* = 0.003

AML, acute myeloid leukemia; ALL, acute lymphoblastic leukemia; aGVHD, acute graft-versus-host-disease; CT, Chemotherapy; cGVHD, chronic graft-versus-host-disease;

FCM, multiparameter flow cytometry; HSCT, hematopoietic stem cell transplantation; HID, haploidentical donor; LFS, leukemia-free survival; MSD, matched sibling donor; MRD, minimal (or measurable) residual disease

### Graft source

In China, 70% of HID and 28% of MSD-HSCT take peripheral blood (PB) plus bone marrow (BM) as stem cell source; among the rest allo-HSCT candidates, PB is predominant stem cell source [1]. Previously, a multicenter study demonstrated that HID-HSCT with mixed grafts of BM + PB achieved longer DFS than PB grafts alone [8]; therefore, mixed grafts were the main component of HID-HSCT (59%) before 2019. Recently, Ma et al. further investigated HID-HSCT with PB alone or mixed grafts of BM and PB (n = 67 vs. 392). The 28-day cumulative incidence of neutrophil and platelet engraftment after HSCT were comparable, with similar rates of grades II–IV aGVHD (29.9% vs. 36.5%, *p* = 0.269), NRM (3.4% vs. 6.9%, *p* = 0.531) and DFS (82.7% vs. 81.3%, *p* = 0.542), suggesting that PB alone might be more effective than mixed grafts of BM + PB [87]. Indeed, due to the COVID-19 pandemic, PB alone has become the predominant graft source [86].

## Recommendation: donor selection and graft source

### General principle of donor selection

1. MSDs should generally be the first choice for allo-HSCT donors.
2. HIDs can be a preferred donor type compared to MSDs for high-risk leukemia; HIDs might be preferred choice for elderly patients with young offspring donors in experienced centers.
3. Patients may undergo URD-HSCT in the absence of MSDs, especially those with nonmalignant hematological diseases who have a lower risk of re-donation for cellular therapy. Additionally, family donors have become more important than URDs during the COVID-19 pandemic [86].
4. CB donors are mainly used for pediatric patients (Figs. 1, 2).

### Algorithm for haploidentical donors

Donors with DSA MFI > 10,000 should be avoided if possible. Desensitization could be applied if no DSA negative donor is available. For HSCT candidates with hematological malignancies, HIDs may be selected considering the following order: children, male siblings, fathers, mismatched siblings (noninherited maternal antigen (NIMA) might be superior to noninherited paternal antigen (NIPA)), mothers, and other collateral relatives. ABO and CMV IgG serological status compatibility between donors and recipients is preferred. A KIR ligand match is preferred for HID-HSCT following the Beijing protocol (Fig. 3).

### Algorithm for unrelated donors

URDs requires HLA matching with high resolution. MUD-HSCT requires 10/10 locus matches for HLA-A, B, C, DRB1, and DQ. Mismatched URDs require at least 8/10 locus matches for HLA-A, B, C, DRB1, and DQ. Donors positive for DSA should also be avoided in mismatched URD-HSCT.

### Algorithm for cord blood donors

The selection of CBDs is based on HLA typing, mononuclear cell (MNC) counts, and primary disease.

For malignant hematological disease patients, ≥ 4/6 loci should be matched, with TNC > (2.5–4.0) × 10^7/kg (recipient weight) and CD34 + cells > (1.2–2.0) × 10^5/kg (recipient weight). For nonmalignant hematological disease patients, ≥ 5/6 loci should be matched, with TNC > 3.5 × 10^7/kg (recipient weight) and CD34 + cells > (1.7 × 10^5/kg (recipient weight) [104].

### General principle of mobilization

G-CSF (5 mg/kg of body weight per day for 5 days) is generally administered to mobilize BM and/or PB cells. The target MNC count is 6–8 × 10^8^/kg recipient weight. Unmanipulated BM (harvested on day 4 after G-CSF administration) and/or PB stem cells (harvested on days 4 and 5 after G-CSF administration) are infused into the recipient on the day of collection. A single dose of pegfilgrastim (12 mg subcutaneously on day 1) is used to mobilize PB; a single dose might avoid the pain of multiple injections [105]. An optimal number of CD34 + cells (≥ 4 × 10^6^ kg) are collected in a single apheresis procedure on day 5.

## Conclusion and perspective

In conclusion, consensus has been updated to reflect the current standard of care and latest available evidence regarding HSCT, especially in China. Randomized prospective controlled trials are lacking for most conditions because transplant decisions are complex. Additionally, new cellular strategies have been developed, potentially changing the situation of allo-HSCT. In summary, periodically updated recommendations will cover the latest cutting-edge developments and improve outcomes in patients undergoing HSCT.

## Data Availability

All data generated or analyzed during this study are included in this published article and its supplementary information files.

## Abbreviations

ALL: Acute lymphoblastic leukemia
allo-HSCT: Allogeneic stem cell transplantation
AML: Acute myeloid leukemia
APL: Acute promyelocytic leukemia
ATG: Anti-thymocyte globulin
BM: Bone Marrow
Car-T: Chimeric antigen receptor (CAR) T-cell
CBMTRG: Chinese Blood and Marrow Transplantation Registry Group
CB: Cord Blood
CIR: Cumulative incidence of relapse
CLL: Chronic lymphocytic leukemia
CML: Chronic myeloid leukemia
CMML: Chronic myelomonocytic leukemia
CR1: First remission
CsA: Cyclosporine
CSH: Chinese Society of Hematology
DFS: Disease-free survival
DIPSS: Dynamic International Prognostic Scoring System
DSAs: Donor-specific anti-HLA antibodies
EBMT: European Cooperative Group for Bone Marrow Transplantation
EFS: Event-free survival
ELN: European Leukemia Net
Fav: Favorable- risk
FCM: Multiparameter flow cytometry
FFS: Failure-free survival
G-CSF: Granulocyte colony-stimulating factor
GRFS: GVHD and relapse-free survival
GVHD: Graft-versus-host-disease
GVL: Graft-vs-leukemia
HCT-CI: Hematopoietic cell transplantation-specific comorbidity index
HIDs: Haploidentical donors
HL: Hodgkin lymphoma
IMDs: Metabolic storage diseases
Int: Intermediate-risk
IPSS: International Prognostic Scoring System
IST: Immunosuppressive therapy
MAC: Myeloablative conditioning
MDS: Myelodysplastic syndrome
MF: Myelofibrosis
MM: Multiple myeloma
MMF: Mycophenolate mofetil
MMR: Major molecular remission
MNC: Mononuclear cell
MPN: Myeloproliferative neoplasms
MRD: Minimal (or measurable) residual disease
MSDs: Matched sibling donors
MTX: Methotrexate
MUDs: Matched unrelated donors
NCCN: National Comprehensive Cancer Network
NIMA: Noninherited maternal antigen
NIPA: Noninherited paternal antigen
NRM: Nonrelapse mortality
OS: Overall survival
PB: Peripheral blood
Ph: Philadelphia Chromosome
PNH: Paroxysmal nocturnal hemoglobinuria
PTCy: Post-transplantation cyclophosphamide
qRT-PCR: Real-time quantitative polymerase chain reaction
R/R: Refractory/relapsed
RIC: Reduced intensity conditioning
SAA: Severe Aplastic anemia
TBI: Total body irradiation
TKIs: Tyrosine kinase inhibitors
TNC: Total nucleated cell
URDs: Unrelated donors
